# Beneficial Effect of COVID-19 Vaccination on Decreased Pulmonary Vascular and Airway Volumes of Patients with Long-COVID Syndrome

**DOI:** 10.3390/medsci14030413

**Published:** 2026-07-22

**Authors:** Eslam Samaha, Emilie Han, Dominika Lukovic, Kevin Hamzaraj, Jutta Bergler-Klein, Ena Hasimbegovic, Mariann Gyöngyösi

**Affiliations:** 1 Department of 1st Internal Medicine, Klinik Donaustadt, 1220 Vienna, Austria; samaha_eslam@hotmail.com; 2 Division of Cardiology, 2nd Department of Internal Medicine, Medical University of Vienna, 1090 Vienna, Austria; emilie.han@meduniwien.ac.at (E.H.); dominika.lukovic@meduniwien.ac.at (D.L.); kevin.hamzaraj@meduniwien.ac.at (K.H.); jutta.bergler-klein@meduniwien.ac.at (J.B.-K.); ena.hasimbegovic@meduniwien.ac.at (E.H.)

**Keywords:** COVID-19, long COVID syndrome, vascular volumes, lung volumes, functional respiratory imaging

## Abstract

Background: Despite normal lung imaging and preserved pulmonary and cardiac function, many patients with long COVID continue to experience persistent respiratory symptoms. This study aimed to quantify the pulmonary blood and airway volumes in patients with long COVID compared with healthy controls and to evaluate the associations of COVID-19 vaccination with these imaging parameters. Methods: Patients with long COVID presenting with persistent respiratory symptoms despite normal laboratory findings, pulmonary function tests, chest radiography, and chest computed tomography (CT) were prospectively enrolled. CT datasets were analyzed using functional respiratory imaging (FRI), incorporating the three-dimensional reconstruction and automated segmentation of the lungs, airways, and pulmonary vasculature. Quantitative imaging parameters were compared with those of historical healthy controls from the COPDGene study, matched for age, sex, body mass index, comorbidities, and pulmonary function test parameters. Results: Thirty patients with long COVID (mean 221 ± 128 days after confirmed SARS-CoV-2 infection) and 30 matched healthy controls were included. Compared with controls, patients with long COVID demonstrated significantly lower pulmonary blood volumes in small and large pulmonary vessels, together with significantly reduced intrapulmonary airway volumes. Within the long COVID cohort, full COVID-19 vaccination was associated with a significantly greater small-vessel pulmonary blood volume and lobar airway volume compared with non-vaccinated individuals. Conclusions: These findings indicate that patients with long COVID exhibit persistent reductions in pulmonary blood and airway volumes despite normal conventional imaging and pulmonary function tests, suggesting the presence of subtle microvascular and small-airway abnormalities that may contribute to ongoing respiratory symptoms. The association between full COVID-19 vaccination and higher small-vessel pulmonary blood and lobar airway volumes suggests a potential protective effect on pulmonary structure and function; however, these findings require confirmation in larger, prospective controlled studies.

## 1. Introduction

The emergence of severe acute respiratory syndrome coronavirus 2 (SARS-CoV-2) caused an unprecedented global health crisis, placing enormous pressure on healthcare systems and substantially advancing our understanding of viral diseases and their long-term sequelae [1]. Although acute coronavirus disease 2019 (COVID-19) primarily affects the respiratory system and, in severe cases, may trigger a systemic hyperinflammatory response, increasing evidence indicates that its consequences frequently extend well beyond the acute phase of infection [2]. Long COVID syndrome, also referred to as post-acute sequelae of SARS-CoV-2 infection (PASC) or post-COVID condition, has consequently emerged as a major clinical and public health challenge, affecting a substantial proportion of individuals after SARS-CoV-2 infection [3,4]. Long COVID is characterized by persistent or newly developed symptoms lasting for weeks or months after the acute infection, involving multiple organ systems [4]. Among these manifestations, respiratory symptoms are among the most common and disabling [5]. Patients frequently report dyspnea, persistent cough, chest pain, and exercise intolerance despite apparent recovery from the acute illness [6]. These persistent symptoms substantially impair functional capacity and quality of life [7]. Growing evidence suggests that pulmonary vascular and airway abnormalities may contribute to the pathophysiology of persistent respiratory symptoms in long COVID. During acute COVID-19, endothelial dysfunction, microvascular thrombosis, small-vessel vasculitis, and dysregulated pulmonary perfusion have been widely reported [8]. In addition, lower respiratory tract involvement, including acute lung injury, pneumonia, pulmonary fibrosis, and, less frequently, bronchiolitis, has been described [9,10]. Chest computed tomography (CT) studies in patients with acute COVID-19 have demonstrated significant reductions in pulmonary blood vessel volumes compared with healthy individuals, and these abnormalities correlate with the severity of hypoxemia and adverse clinical outcomes [11]. Whether similar pulmonary vascular and airway abnormalities persist in patients with long COVID who have normal conventional chest imaging and pulmonary function tests remains largely unknown.

More recently, photon-counting detector CT identified subtle pulmonary abnormalities, including ground-glass opacities and linear bands, in a small cohort of patients with long COVID [12]. Nevertheless, many patients with persistent respiratory symptoms, particularly dyspnea and exertional breathlessness, have normal pulmonary function tests and no detectable abnormalities on conventional chest CT. These findings suggest that conventional imaging may lack sufficient sensitivity to detect subtle alterations in the distal airways and pulmonary microvasculature. Therefore, the present study aimed to re-evaluate chest CT scans with normal conventional findings in symptomatic patients with long COVID using functional respiratory imaging (FRI; Fluidda^®^, Kontich, Belgium). This advanced post-processing technique enables the quantitative three-dimensional assessment of the pulmonary vascular and airway volumes, allowing the detection of subtle structural alterations beyond routine radiological evaluation. Quantitative FRI parameters were compared with those of matched healthy controls to identify potential imaging biomarkers of long COVID.

In addition, patients were stratified into fully vaccinated, partially vaccinated, and non-vaccinated groups according to the definitions of the Centers for Disease Control and Prevention to investigate the association between the COVID-19 vaccination status and pulmonary vascular and airway volumes. This analysis was based on evidence that COVID-19 vaccination attenuates the severity of acute SARS-CoV-2 infection and reduces the risk of developing long COVID [13,14], raising the possibility that vaccination may also preserve pulmonary vascular and airway integrity following infection.

## 2. Results

The baseline characteristics of the study population (n = 60) are summarized in Table 1. The long COVID group comprised 30 participants enrolled from our prospective cohort study. Data for the healthy control group were obtained from the COPDGene study. Because the minimum age of participants in the COPDGene healthy control cohort was 45 years, the mean age of the control group was inevitably higher than that of the long COVID group. However, this difference was not statistically significant. The pulmonary function test results did not differ significantly between long COVID patients and controls (Table 1).

The current medications used by the long COVID cohort are summarized in Table 2. The most frequently prescribed medications were antihistamines and beta-blockers. In addition, several patients were treated with bronchodilators for symptomatic relief despite having normal pulmonary function test results and no evidence of underlying structural lung disease.

In the long COVID group, the mean time from the first SARS-CoV-2 infection to clinical presentation was 221 ± 128 days, and lung CT imaging was performed 72 ± 61 days after the initial outpatient visit. Twenty-three patients (76.7%) underwent high-resolution CT, while seven (23.3%) underwent spiral CT scanning. In the control group, all participants (100%) underwent high-resolution CT imaging. Conventional CT evaluation revealed no pathological findings in any participant, except for two individuals (6.7%) in the long COVID group, who exhibited minimal, non-specific changes in the form of isolated hilar lymphadenopathy.

All CT datasets were subsequently analyzed using FRI to quantify the pulmonary airway and vascular volumes. The following airway parameters were assessed: the intrapulmonary airway volume (IVAW, mL) and the intrapulmonary lobar airway volume (IVLOBE, L), representing the volumes of the intrapulmonary airways, including the main bronchi and bronchioles. Pulmonary vascular volumes were quantified as BV5 (pulmonary blood vessel volume in vessels with a cross-sectional area of 1.25–<5 mm^2^), BV5–10 (vessels with a cross-sectional area of ≥5–<10 mm^2^), and BV10–140 (vessels with a cross-sectional area of ≥10–140 mm^2^). All pulmonary vascular volumes were expressed in milliliters (mL).

Table 3 summarizes the age-adjusted differences in pulmonary blood volumes and airway volumes between patients with long COVID and healthy controls. Pulmonary blood volumes were assessed in vessels with cross-sectional areas up to 140 mm^2^, corresponding to an estimated vessel diameter of approximately 13.5 mm. After adjustment for age, the pulmonary blood volumes in both small and larger vessels were significantly lower in the long COVID group than in the control group. In addition, both the IVAW and IVLOBE were significantly reduced in patients with long COVID. Supplementary Table S1 presents the age-adjusted mean differences between groups, β regression coefficients, and the results of the homogeneity of variance assessment (Levene’s test). Further adjustment for additional potential confounders, including sex, comorbidities, and pulmonary function test parameters, did not materially alter these findings, and the differences between the groups remained statistically significant.

### Effects of Vaccination Status on FRI Parameters in Long COVID Patients

Nine patients were fully vaccinated, ten were partially vaccinated, and eleven were non-vaccinated at the time of lung CT imaging. The first COVID-19 vaccination was administered at a mean of 163 ± 150 days after the initial SARS-CoV-2 infection, in accordance with contemporaneous recommendations suggesting a minimum interval of six months between infection and vaccination. In all fully and partially vaccinated patients, lung CT imaging was performed after vaccination. Table 4 lists the demographic, lung functional test, and quality of life questionnaire data of the long COVID subgroups. In addition, medication use did not differ significantly among the three vaccination groups.

Blood anti-SARS-CoV-2 spike antibody titers were significantly higher in fully vaccinated patients than in non-vaccinated patients. The VAS self-reported health score was significantly higher in the fully vaccinated subgroup than in the partially vaccinated and non-vaccinated subgroups, indicating a better perceived health status. In addition, the exercise-induced dyspnea score was significantly lower in fully vaccinated than in non-vaccinated patients.

Laboratory parameters are summarized in Table 5. No significant differences were observed among the three long COVID vaccination subgroups, and all measured laboratory values were within the normal reference ranges.

**Figure 1:**
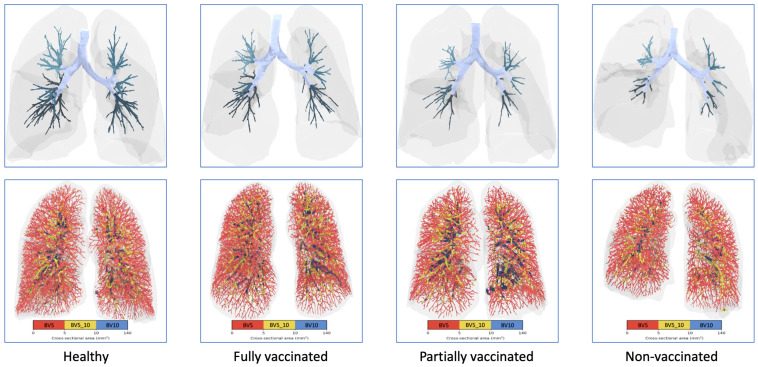
Functional respiratory imaging (FRI) of the airways and pulmonary vasculature of healthy, fully, or partially or non-vaccinated long COVID individuals. Upper panels: FRI images of the bronchus tree, demonstrating the intrapulmonary volume of the airway (IVAW). Bottom panels: FRI display of the pulmonary vessels (divided into small (red), medium-sized (yellow), and larger vessels (blue). In long COVID patients, smaller airway volumes and decreased total pulmonary blood volumes, as well as decreased blood volumes of the small vessels, are observed.

A representative example of FRI is shown in Figure 1, illustrating the pulmonary vasculature segmented into small (red), medium-sized (yellow), and large (blue) vessels, as well as the distribution and localization of the bronchi and bronchioles, in a healthy control and in fully vaccinated, partially vaccinated, and non-vaccinated patients with long COVID.

**Figure 2:**
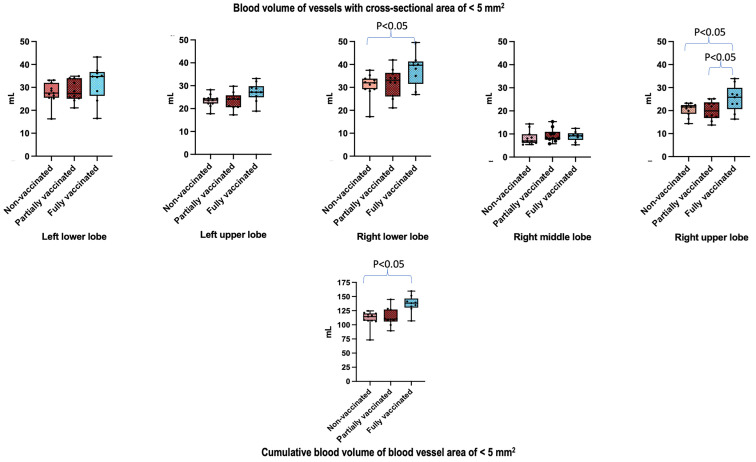
Blood volumes of vessels with a cross-sectional area of 1.25–<5 mm^2^ (corresponding to small–medium-sized pulmonary muscular arteries and medium-sized pulmonary veins) in non-vaccinated (n = 11), partially vaccinated (n = 10), and fully vaccinated (n = 9) long COVID patients.

Fully vaccinated patients with long COVID exhibited significantly greater pulmonary blood vessel volumes in small vessels with cross-sectional areas <5 mm^2^ compared with partially vaccinated and non-vaccinated patients (Figure 2; Supplementary Table S2).

**Figure 3:**
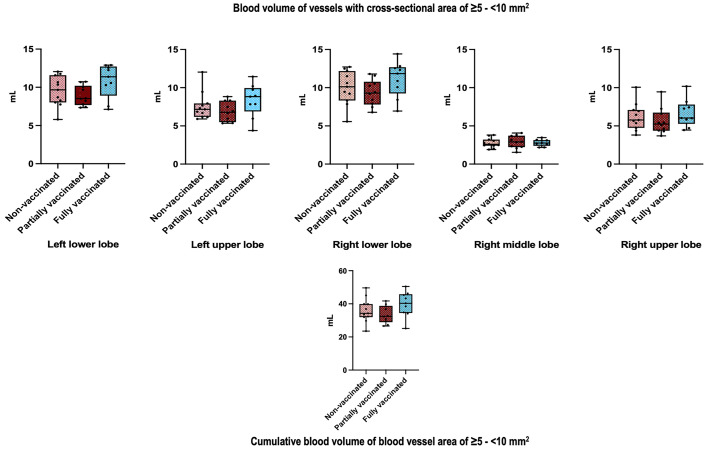
Blood volumes of vessels with a cross-sectional area of ≥5–<10 mm^2^ (corresponding to large elastic segmental pulmonary arteries and medium-sized pulmonary veins) in non-vaccinated (n = 11), partially vaccinated (n = 10), and fully vaccinated (n = 9) long COVID patients.

Only minor differences were observed in medium-sized vessels, corresponding to large elastic segmental pulmonary arteries and medium-sized pulmonary veins (Figure 3).

**Figure 4:**
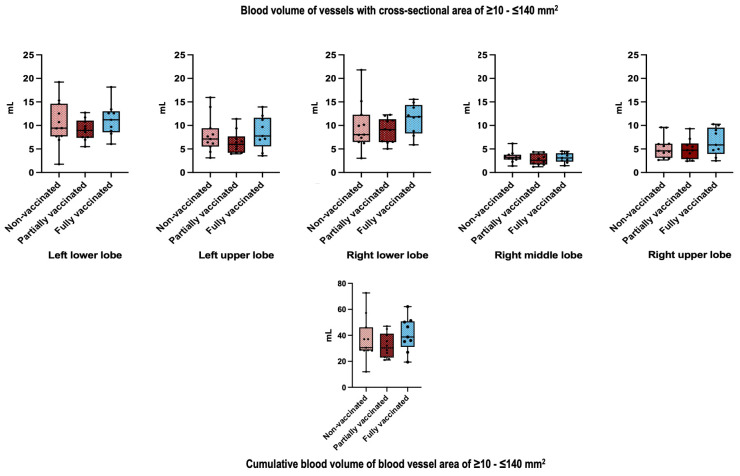
Blood volumes of vessels with a cross-sectional area of ≥10 and 140 mm^2^ (corresponding to large elastic segmental pulmonary arteries and large pulmonary veins) in non-vaccinated (n = 11), partially vaccinated (n = 10), and fully vaccinated (n = 9) long COVID patients.

Similarly, no significant differences were detected among the vaccination groups with respect to the blood volumes in the large pulmonary arteries and veins (Figure 4).

**Figure 5:**
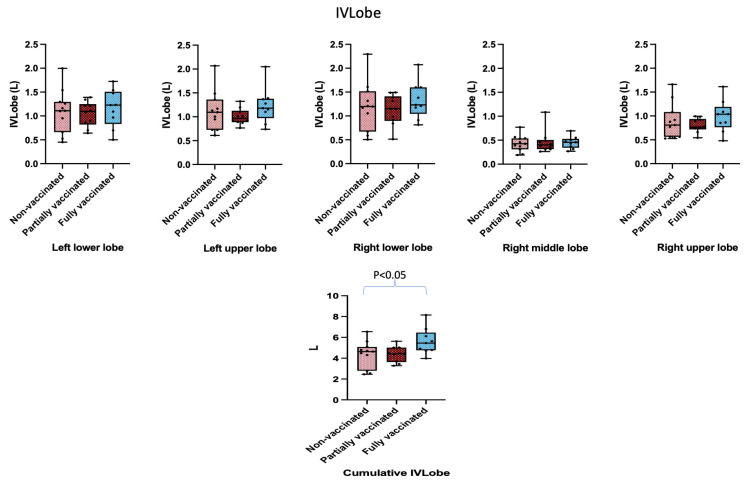
Intrapulmonary airway volumes (IVLOBEs) of the 5 lobar segments, as well as the cumulative total lobar volume, in non-vaccinated (n = 11), partially vaccinated (n = 10), and fully vaccinated (n = 9) long COVID patients. A significantly higher cumulative IVLOBE can be seen in the fully vaccinated long COVID patients compared to partially or non-vaccinated individuals (bottom panel).

In terms of airway parameters, the IVLOBE tended to be higher in fully vaccinated patients across all lung lobes, resulting in a significantly greater cumulative IVLOBE compared with partially vaccinated and non-vaccinated individuals (Figure 5).

**Figure 6:**
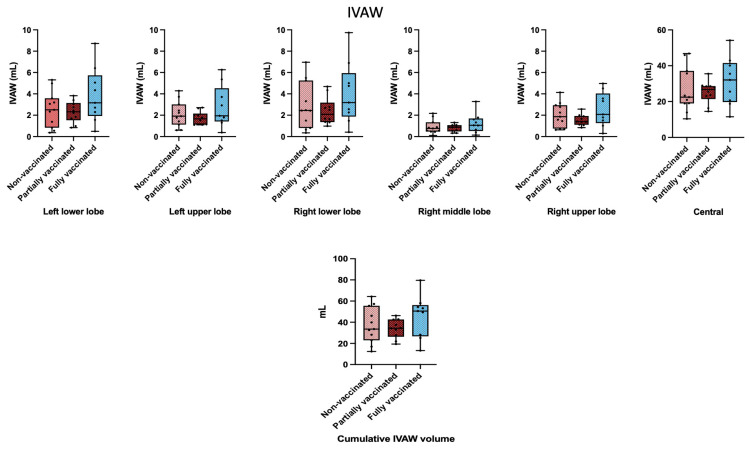
Intrapulmonary volume of airways (IVAW) in all 5 lobar segments and the central pulmonary area, with the cumulative IVAW in non-vaccinated (n = 11), partially vaccinated (n = 10), and fully vaccinated (n = 9) long COVID patients. No significant difference was found between the three long COVID groups.

The IVAW was also higher in the fully vaccinated group; however, this difference did not reach statistical significance (Figure 6).

In the multivariable analysis with BV5 TOTAL as the dependent variable and the vaccination subgroup as the main explanatory variable, adjusted for age and sex, the model explained 26.3% of the variance in BV5 TOTAL (R^2^ = 0.263; adjusted R^2^ = 0.145). Although the overall model did not reach statistical significance (F(4, 25) = 2.23, *p* = 0.095), the vaccination subgroup showed a borderline association with BV5 TOTAL (F(2, 25) = 3.09, *p* = 0.063). Compared with fully vaccinated patients, non-vaccinated patients had significantly lower BV5 TOTAL values (β = −19.6 mL, 95% CI −36.2 to −3.0; *p* = 0.023), whereas the difference between partially and fully vaccinated patients did not reach statistical significance (β = −15.9 mL, 95% CI −34.1 to 2.3; *p* = 0.084). Neither age nor sex was independently associated with BV5 TOTAL.

Comparable analyses were performed for BV5 RUL (right upper lobe), BV5 RLL (right lower lobe), and IVLOBE TOTAL. Although similar trends were observed, the vaccination subgroup was not significantly associated with BV5 RUL (*p* = 0.142), BV5 RLL (*p* = 0.079), or IVLOBE TOTAL (*p* = 0.078).

Variables that differed significantly among the vaccination subgroups in the univariate analyses (VAS score, exercise-induced dyspnea score, and anti-spike antibody level) were subsequently included in an exploratory sensitivity analysis together with the vaccination subgroup, age, and sex to assess whether the observed association between vaccination status and BV5 TOTAL was independent of these clinical characteristics. After additional adjustment, the association between the vaccination subgroup and BV5 TOTAL remained essentially unchanged (overall *p* = 0.065), while none of the additional covariates was independently associated with BV5 TOTAL. Compared with fully vaccinated patients, non-vaccinated patients continued to exhibit substantially lower BV5 TOTAL values (β = −34.4 mL, 95% CI −63.2 to −5.5 mL), indicating that the observed association was robust to adjustment for these potential confounders.

## 3. Discussion

This study demonstrates for the first time that patients with long COVID who report persistent pulmonary symptoms despite normal pulmonary function tests and conventional chest CT findings exhibit significant alterations in pulmonary structure when assessed using FRI. Specifically, these patients showed reduced pulmonary blood volumes in small-to-medium-sized pulmonary muscular arteries and medium-sized pulmonary veins, as well as in large elastic segmental pulmonary arteries and large pulmonary veins. In addition, the IVAW and IVLOBE were significantly reduced compared with healthy control subjects. To account for potential confounding by age, all FRI parameters were analyzed using ANCOVA with age included as a covariate. After age adjustment, the differences in BV5, BV10–140, IVAW, and IVLOBE between long COVID patients and controls remained statistically significant. Furthermore, age was not an independent predictor of any of these FRI parameters, indicating that the observed reductions in the pulmonary blood and airway volumes cannot be explained by differences in age between the study groups. These findings support the conclusion that the observed alterations are associated with long COVID rather than age-related physiological differences.

Moreover, fully vaccinated patients with long COVID demonstrated more favorable pulmonary physiology than partially vaccinated or non-vaccinated individuals, characterized by significantly greater pulmonary blood volumes in small-to-medium–sized pulmonary muscular arteries and medium-sized pulmonary veins, as well as a higher cumulative IVLOBE. The multivariable analysis demonstrated a borderline association between vaccination status and the FRI parameters after adjustment for age and sex. Although the overall effect of the vaccination subgroup did not reach conventional statistical significance, non-vaccinated patients had significantly lower BV5 TOTAL values than fully vaccinated patients. These findings suggest a potential protective association of COVID-19 vaccination with the preservation of the pulmonary microvasculature, possibly through the attenuation of vascular inflammation, microthrombus formation, and chronic bronchiolar involvement. However, given the limited sample size and observational study design, these findings should be interpreted with caution. Larger prospective studies are required to confirm these associations, establish causality, and elucidate the underlying biological mechanisms.

Fully vaccinated patients with long COVID had significantly higher VAS scores (0–100 scale, with higher scores indicating better perceived health status) and reported less exercise-induced dyspnea than non-vaccinated patients, consistent with a better overall health status. Because the VAS score, exercise-induced dyspnea, and anti-spike antibody levels may represent clinical or immunological consequences of vaccination rather than true baseline confounders, the primary multivariable models were adjusted for age and sex only to elaborate on the effect of vaccination status on the FRI parameter. These additional variables were subsequently included in exploratory sensitivity analyses to assess the robustness of the findings. None of these variables was independently associated with BV5 TOTAL, BV5 RUL, BV5 RLL, or IVLOBE TOTAL, and their inclusion did not materially alter the associations between the vaccination subgroup and the FRI parameters.

A particularly noteworthy finding of this study is that all observed pulmonary abnormalities were detectable exclusively by FRI, despite the absence of pathological findings on routinely interpreted clinical thoracic CT scans. While standard CT imaging primarily depicts structural and morphological sequelae, FRI adds a complementary quantitative and functional dimension by characterizing regional airflow limitation, vascular distribution, and ventilation heterogeneity—features that static imaging may fail to capture [15,16,17,18]. Integrating FRI-derived functional metrics with established CT-based phenotypes may therefore improve the precision of post-COVID pulmonary assessment, particularly in patients whose radiological findings do not adequately explain ongoing respiratory complaints.

Recent studies have demonstrated the ability of FRI to identify region-specific quantitative structural and functional lung abnormalities associated with disease severity, air trapping, and airflow limitation in chronic obstructive pulmonary disease, asthma, or cystic fibrosis [15,16,17,19,20]. These FRI-derived abnormalities correlate with clinical outcomes and enable discrimination between treatment responders and non-responders, offering greater sensitivity than global pulmonary function measures such as FEV_1_ and improving the monitoring of disease progression and therapeutic response [15,18]. By facilitating refined phenotyping and the detailed characterization of pathophysiological changes, FRI supports individualized treatment strategies and provides deeper insight into regional lung dysfunction across chronic airway diseases [15,16,17]. Given the large number of individuals affected by long COVID worldwide, the wider availability of FRI could facilitate the objective assessment of persistent pulmonary symptoms in patients with long COVID. Furthermore, FRI may help to identify patients who could potentially benefit from targeted therapies, such as bronchodilator or anticoagulant treatment. However, prospective studies are required to establish its clinical utility and role in guiding therapeutic decision-making in long COVID patients.

In healthy individuals, small pulmonary vessels—including arterioles, capillaries, and venules—constitute the largest proportion of the pulmonary blood volume [21,22]. Alterations in pulmonary blood distribution, as observed in conditions such as chronic obstructive pulmonary disease and acute respiratory distress syndrome, are thought to result from chronic vascular remodeling and the loss of small vessels [23,24]. During acute SARS-CoV-2 infection, patients with severe respiratory involvement have been shown to exhibit a markedly reduced proportion of blood flow within small pulmonary vessels [25]. These vascular changes have been associated with increased oxygen requirements, prolonged hospitalization, and a greater need for intubation and risk of mortality [11].

In the present study, we focused on patients with mild to moderate COVID-19 who did not require hospitalization during the acute phase but continued to experience persistent pulmonary symptoms months after infection. Despite normal findings on conventional imaging and pulmonary function testing, these patients exhibited significantly reduced intrapulmonary airway volumes (IVAW and IVLOBE), as well as decreased pulmonary blood volumes in both small and large vessels. These findings suggest that lingering respiratory symptoms in long COVID may be driven by subtle, functionally relevant abnormalities in pulmonary microcirculation and airway architecture. Potential mechanisms include abnormal vasoconstriction, endothelial inflammation, and microthrombotic processes within the pulmonary vasculature, leading to impaired alveolar–capillary gas exchange or peribronchiolar edema—changes that may remain undetectable on standard thoracic imaging modalities [25,26,27,28,29].

In parallel, chest CT studies and systematic reviews have consistently reported residual parenchymal abnormalities—such as ground-glass opacities, reticular patterns, traction bronchiectasis, and fibrotic-like changes—persisting for several months after acute infection, particularly among patients with severe initial disease [30,31]. Meta-analyses further demonstrate a substantial pooled prevalence of long-term CT abnormalities and suggest that both structural and functional pulmonary recovery may remain incomplete for months or even years following SARS-CoV-2 infection [32].

Furthermore, pulmonary perfusion abnormalities may represent potential targets for tailored therapeutic strategies in patients with long COVID. The identification of such abnormalities could help to stratify patients who may benefit from vasodilatory or antithrombotic interventions.

### Limitations

This study has several limitations. First, its retrospective design and the relatively small sample size limit the generalizability of the findings. In addition, only a subset of patients with diagnosed long COVID underwent chest CT imaging with the required slice thickness of ≤1 mm, which was necessary for the FRI analysis. Nevertheless, only participants with complete datasets were included to ensure data integrity and analytical robustness.

Second, pre-COVID-19 chest CT scans were not available, as none of the patients had thoracic symptoms prior to SARS-CoV-2 infection. Consequently, intraindividual comparisons before and after COVID-19 could not be performed. However, given the absence of pathological findings on post-infection CT imaging, it is reasonable to assume that pre-infection CT findings would likewise have been normal.

Although the observed reductions in BV5 and airway volumes are consistent with pulmonary microvascular dysfunction, endothelial injury, microthrombus formation, and impaired alveolar–capillary gas exchange, our study did not include direct assessments of these mechanisms. Specifically, pulmonary perfusion imaging, biomarkers of endothelial activation, gas exchange measurements, objectively assessed exercise capacity, cardiopulmonary exercise testing, and pulmonary diffusion capacity were not available. These investigations were not included in the Ethics Committee-approved study protocol and could not be performed during routine outpatient care. Therefore, the proposed pathophysiological mechanisms remain speculative and should be confirmed in future prospective studies incorporating comprehensive physiological and functional assessments.

The majority of patients with long COVID (76%) underwent high-resolution chest CT, whereas the remaining patients were examined using alternative CT acquisition protocols. Regardless of the acquisition protocol, all CT datasets fulfilled the technical requirements for FRI analysis, including a slice thickness of ≤1 mm, volumetric thin-slice DICOM images, full inspiratory acquisition, complete lung coverage, and the absence of relevant motion artifacts. According to the FRI methodology, these technical requirements are essential to ensure reliable quantitative analysis. Once these criteria are met, the software standardizes CT datasets obtained using different acquisition protocols, enabling a comparable quantitative assessment of airway and pulmonary vascular parameters. Therefore, differences in CT acquisition protocols are unlikely to have influenced the FRI measurements in the present study.

Functional respiratory imaging (FRI; Fluidda NV, Kontich, Belgium) is a validated quantitative CT-based image analysis platform that combines the automated three-dimensional segmentation of the airways, lungs, and pulmonary vasculature with computational fluid dynamics to derive regional structural and functional biomarkers. The technology has undergone extensive technical, clinical, and regulatory validation [33,34,35]; has received clearance from the U.S. Food and Drug Administration (FDA) as a clinical software platform (Broncholab) to support the diagnosis and monitoring of respiratory diseases; and has been widely applied in studies of respiratory disorders, including chronic obstructive pulmonary disease, asthma, cystic fibrosis, pulmonary hypertension, and COVID-19-related lung disease [15,36,37]. In addition, FRI has been used in numerous interventional studies to evaluate the effects of inhaled therapies, including aerosolized medications and inhaled pulmonary vasodilators, on the regional pulmonary vascular morphology and lung function (e.g., ClinicalTrials.gov identifiers: NCT04876677 and NCT01684384). Unfortunately, this postprocessing imaging software is only limitedly available due to financial factors.

Furthermore, the FRI software does not distinguish between arterial and venous pulmonary blood volumes—a limitation that may be clinically relevant when considering targeted therapeutic strategies, particularly those involving anticoagulation. Future methodological advances allowing the separation of arterial and venous compartments could enhance the clinical applicability of FRI-derived perfusion metrics.

From a technical perspective, the automated FRI software is unable to differentiate pulmonary blood volumes within the smallest vascular compartments where gas exchange primarily occurs, including pulmonary arterioles (diameter 0.04–0.1 mm; cross-sectional area < 0.5 mm^2^), capillaries (diameter 0.005–0.01 mm; cross-sectional area < 0.0005 mm^2^), and venules (diameter 0.02–0.1 mm; cross-sectional area < 0.05 mm^2^). Nevertheless, we observed significantly reduced pulmonary blood volumes within the smallest measurable vascular range (cross-sectional area < 5 mm^2^) in patients with long COVID, particularly among partially vaccinated and non-vaccinated individuals, suggesting clinically relevant microvascular involvement despite these technical constraints.

Furthermore, FRI quantifies the pulmonary blood volume only in vessels with cross-sectional areas up to 140 mm^2^ (corresponding to an estimated vessel diameter of approximately 13.5 mm), encompassing large elastic segmental pulmonary arteries and large pulmonary veins. The main pulmonary artery (approximately 15–20 mm in diameter) and main pulmonary veins (approximately 10–15 mm) exceed this range. Consequently, the pulmonary blood volumes reported in this study represent an underestimation of the total pulmonary blood volume.

Additional limitations should be acknowledged. The number of control subjects was relatively small, and only limited clinical data were available for this group, as the controls were included from an external historical database and restricted to variables required for matching, in accordance with patient confidentiality and ethical regulations.

## 4. Materials and Methods

### 4.1. Study Design

This study is a retrospective analysis of adult patients with long COVID derived from a prospective cohort study. The study was approved by the local Ethics Committee and conducted in accordance with the Declaration of Helsinki. In addition, the prospective registry was extended with a historical case–control cohort from the COPDGene study (NCT00608764), which included healthy individuals. Controls were matched to long COVID patients based on age, sex, race, body mass index (BMI), comorbidities (current smoking status, arterial hypertension, hypercholesterolemia, coronary artery disease, diabetes mellitus, chronic obstructive pulmonary disease, stroke, congestive heart failure, and chronic kidney disease), and pulmonary function test parameters, including forced vital capacity (FVC), forced expiratory volume in 1 s (FEV_1_), and peak expiratory flow (PEF). Permission to include the control cohort was obtained from FLUIDDA (FLUIDDA^®^, Kontich, Belgium) [38] to enable the comparison of pulmonary anatomical and functional parameters between long COVID patients and control individuals using the FRI software. The study design, data analysis, and reporting followed the Strengthening the Reporting of Observational Studies in Epidemiology (STROBE) guidelines.

### 4.2. Long COVID Patient Cohort

Between 1 March 2020 and 9 September 2021, consecutive patients with a prior quantitative real-time PCR-confirmed SARS-CoV-2 infection (delta variant) who presented to our long COVID cardiac outpatient clinic were enrolled in a prospective registry (POSTCOV Registry; Ethics Committee numbers: 1008/2021, 2269/2021, and 1758/2022; ClinicalTrials.gov identifier: NCT05398952). Only patients with mild to moderate COVID-19 who did not require hospitalization during the acute phase of infection were included. Additional inclusion criteria for this substudy of the POSTCOV Registry were the presence of symptoms affecting at least three organ systems, fulfilling the definition of multiorgan involvement and long COVID syndrome [16,17,18], as well as new-onset pulmonary symptoms following SARS-CoV-2 infection. These respiratory symptoms included resting or exertional dyspnea, cough, or chest pain, as documented by the COVID-19 Yorkshire Rehabilitation Screening (C19-YRS) and EQ-5D-3L questionnaires. Eligible patients were required to have normal pulmonary function test results and no abnormalities on chest radiography or lung CT. Exclusion criteria included hospitalization during acute SARS-CoV-2 infection; pre-existing pulmonary disease; acute inflammatory conditions or infections; and systemic diseases—such as rheumatic disorders, systemic or pulmonary thromboembolic events, or hematologic or malignant diseases—that could affect pulmonary structure or function. Patients were categorized as fully vaccinated (two primary vaccine doses plus one updated booster dose), partially vaccinated (one or two primary vaccine doses), or non-vaccinated at the time of lung CT imaging, according to the Centers for Disease Control and Prevention definitions (www.cdc.gov). Written informed consent was obtained from all participants prior to inclusion in the registry and this substudy. The time interval between the most recent onset of COVID-19 and the outpatient clinical visit and the waiting time for CT were recorded for each patient. Clinical data—including age, sex, race, body mass index (BMI), comorbidities (smoking status, arterial hypertension, hypercholesterolemia, peripheral vascular disease, coronary artery disease, diabetes mellitus, and chronic kidney disease), socioeconomic status, number of infections, viral variants, baseline symptom severity, socioeconomic factors, healthcare-seeking behavior, pulmonary function test parameters, and current medication were recorded for all long COVID participants.

### 4.3. Laboratory Investigations

Routine laboratory data were available only for patients with long COVID. Hematological and general organ function parameters—including hemoglobin concentrations, platelet and leukocyte counts, serum albumin, creatinine, aspartate aminotransferase (AST/SGOT), alanine aminotransferase (ALT/SGPT), thyroid-stimulating hormone (TSH), and serum iron—were collected. Coagulation parameters included the prothrombin time, international normalized ratio (INR), activated partial thromboplastin time (aPTT), and von Willebrand factor (vWF). Cardiac biomarkers, including troponin T, creatine kinase (CK), and N-terminal pro-brain natriuretic peptide (NT-proBNP), as well as inflammatory markers—C-reactive protein (CRP), lactate dehydrogenase (LDH), ferritin, transferrin, transferrin saturation, interleukin-6 (IL-6), and procalcitonin—were measured in all long COVID patients. All laboratory analyses were performed according to the standardized protocols of the Department of Laboratory Medicine, as published on the institutional website (https://www.akhwien.at/default.aspx?pid=3985, accessed on 15 January 2025).

### 4.4. Lung CT

Thoracic CT was performed in patients reporting post-COVID pulmonary symptoms at the Department of Biomedical Imaging and Image-Guided Therapy, Vienna General Hospital. Conventional clinical chest CT scans using energy-integrating detector technology were acquired as previously described [12] (Supplementary Methods). Normal CT findings were defined by the absence of pulmonary abnormalities according to the criteria proposed by Solomon et al. [39] and the Fleischner Society glossary of thoracic imaging terminology [40]. Specifically, normal scans showed no evidence of bronchiectasis, bronchiolectasis, bronchial wall thickening, air trapping, consolidation, ground-glass opacities, honeycombing, linear bands, fibrosis, mosaic attenuation, pleural thickening, reticulation, volume loss, emphysema, enlarged lymph nodes, or signs of acute or chronic infection.

### 4.5. FRI

Chest CT scans of long COVID patients with normal diagnostic findings and good technical quality were prospectively selected. Patient CT data in DICOM format, with all personal health information removed, were transferred to FLUIDDA (Kontich, Belgium) via a secure box solution (Dropbox, Box) and PACS integration with Ambra Health. For a successful analysis, the requirements for the CT scans were good quality and a consistent slice thickness of 1 mm or smaller.

FRI data collected from the scans were processed and analyzed using the proprietary FLUIDDA software, performed at the company site by a software expert, due to license restrictions. This included the following steps: (1) lung and airway structures were segmented from the images to create anatomical models of the respiratory system; (2) respiratory dynamics, such as lung volume changes, were calculated based on the captured data; (3) airway dimensions, including the diameter, cross-sectional area, and branching patterns, were analyzed. Accordingly, the following parameters were extracted for each patient using automated processes with manual checks and corrections if needed:

- Airway volumes were expressed as the intrapulmonary airway volume (IVAW, mL) and intrapulmonary lobar airway volume (IVLOBE, L) in the main bronchi and bronchioles;
- Pulmonary vessel volumes were expressed as BV5 (pulmonary vascular volume in vessels between 1.25 and <5 mm^2^ in cross-sectional area, corresponding to small–medium-sized pulmonary muscular arteries and medium-sized pulmonary veins), BV5–10 (pulmonary vascular volume in vessels between ≥5 and <10 mm^2^ in cross-sectional area, corresponding to large elastic segmental pulmonary arteries and medium-sized pulmonary veins), and BV10 (pulmonary vascular volume in vessels between ≥10 and 140 mm^2^ in cross-sectional area, corresponding to large elastic segmental pulmonary arteries and large pulmonary veins). Both the airway and vessel volumes were calculated in all five lung lobes (left upper lobe (LUL), left lower lobe (LLL), right upper lobe (RUL), right middle lobe (RML), and right lower lobe (RLL)), as well as the upper lobes (UL) and lower lobes (LL).

### 4.6. Statistical Analysis

Continuous variables with a normal distribution are presented as the mean ± standard deviation (SD), whereas skewed continuous variables are reported as medians with interquartile ranges (IQRs). Categorical variables are expressed as frequencies and percentages.

To determine whether the observed differences in the pulmonary blood and airway volumes between the long COVID and control groups were independent of age, an analysis of covariance (ANCOVA) was performed. Group (long COVID vs. control) was included as a fixed factor, and age was entered as a continuous covariate. Adjusted group means (estimated marginal means) with 95% confidence intervals (CIs) were calculated, and pairwise comparisons were performed using the Bonferroni correction. The assumption of homogeneity of variances was assessed using Levene’s test.

To evaluate the robustness of the findings, additional ANCOVA models were constructed with further adjustment for sex, comorbidities, and pulmonary function test parameters.

Comparisons among the fully vaccinated, partially vaccinated, and non-vaccinated long COVID subgroups were performed using one-way analysis of variance (ANOVA) for normally distributed continuous variables, followed by Bonferroni-adjusted post hoc tests to account for multiple comparisons. Non-normally distributed continuous variables were analyzed using the Kruskal–Wallis test. Categorical variables were compared using Pearson’s χ^2^ test. Multivariable analyses were performed to evaluate whether the associations between vaccination status and the significant FRI parameters were independent of potential confounding variables.

Because of the limited sample size, the multivariable models were restricted to a small number of prespecified covariates to minimize the risk of model overfitting. Separate analysis of covariance (ANCOVA) models were constructed for each significant continuous FRI endpoint (BV5 RLL, BV5 RUL, BV5 TOTAL, and IVLOBE TOTAL), with the vaccination subgroup included as the fixed factor and age and sex entered as covariates. To assess the robustness of the findings, sensitivity analyses were performed by additionally adjusting for variables that differed significantly among the vaccination subgroups, including demographic characteristics, laboratory parameters, and patient-reported outcome measures.

All statistical analyses were conducted using the SPSS software 30.0.0.0 (172); IBM Corp., Armonk, NY, USA). A two-sided *p* value of <0.05 was considered statistically significant.

## 5. Conclusions

Our study found that patients with long COVID demonstrated significantly lower pulmonary blood volumes in small and large pulmonary vessels compared with controls. The intrapulmonary airway volumes were significantly reduced in the long COVID group. The mechanisms underlying long COVID-related pulmonary dysfunction are complex and likely involve persistent inflammation, microvascular injury, microthrombosis, and bronchiolar involvement, including edema or bronchiolitis. Pathological alterations within the pulmonary microcirculation, resulting in impaired alveolar–capillary gas exchange, may represent a central pathophysiological mechanism driving the persistent respiratory symptoms observed in patients with long COVID. In our cohort, full vaccination was associated with the partial attenuation of these microvascular and airway abnormalities.

## Figures and Tables

**Table 1:** Baseline characteristics (matching parameters).

Variable	Long COVID (n = 30)	Control (n = 30)
Male, n (%)	11 (36.7%)	11 (36.7%)
Age (years)	44.9 ± 12.6	54.8 ± 6.8
Race White, n (%)	30 (100%)	28 (93.3%)
BMI (kg/m^2^)	25.9 ± 4.3	26.3 ± 2.7
Comorbidities, n (%)		
Chronic Obstructive Pulmonary Disease, n (%)	0 (0%)	0 (0%)
Current Smoker, n (%)	3 (10.0%)	0 (0%)
Arterial Hypertension, n (%)	6 (20.0%)	6 (20.0%)
Hypercholesterolemia, n (%)	8 (26.7%)	8 (26.7%)
Peripheral Vascular Disease, n (%)	0 (0%)	0 (0%)
Non-Significant Coronary Artery Disease, n (%)	2 (6.7%)	0 (0%)
Diabetes Mellitus, n (%)	1 (3.3%)	3 (10.0%)
Chronic Kidney Disease, n (%)	0 (0%)	0 (0%)
Stroke, n (%)	0 (0%)	0 (0%)
Congestive Heart Failure, n (%)	0 (0%)	0 (0%)
PFT, n (%)		
FVC (L)	3.72 ± 0.90	3.93 ± 0.7
FEV1 (L)	2.89 ± 0.74	3.04 ± 0.58
PEF (L/s)	7.69 ± 1.39	7.81 ± 1.57

BMI: body mass index, PFT: pulmonary function test, FVC: forced vital capacity, FEV1: forced expiratory volume in 1 s, PEF: peak expiratory flow. Continuous parameters are reported as mean ± standard deviation, and nominal data are reported as frequencies with percentages (%).

**Table 2:** Medical treatment in the long COVID group.

Medication n (%)	Long COVID (n = 30)
Bronchodilator, n (%)	5 (16.7%)
NSAID, n (%)	5 (16.7%)
ARB, n (%)	1 (3.3%)
ACE inhibitor, n (%)	0 (0%)
Beta-blocker, n (%)	4 (13.3%)
Aldosterone antagonist, n (%)	2 (6.7%)
H2-receptor blocker, n (%)	7 (23.3%)
Statin, n (%)	3 (10%)
Metformin, n (%)	1 (3.3%)

NSAID: non-steroidal anti-inflammatory drug; ARB: angiotensin receptor antagonist; ACE: angiotensin-converting enzyme.

**Table 3:** Functional respiratory imaging parameters in long COVID patients and controls.

Age-Adjusted FRI Parameter	Long COVID (n = 30)	Control (n = 30)	*p* Value
Pulmonary blood volume			
BV5 (mL) mean (95% CIs)	117.2 (108.9; 125.5)	132.8 (124.5; 141.1)	0.015
BV5–10 (mL) mean (95% CIs)	36.2 (33.7; 38.7)	35.7 (33.2; 38.2)	0.799
BV10–140 (mL) mean (95% CIs)	36.0 (31.1; 41.0)	63.3 (58.4; 68.3)	<0.001
Pulmonary airway volume			
IVAW (mL) mean (95% CIs)	37.7 (31.1; 44.4)	52.8 (46.1; 59.4)	0.004
IVLOBE (L) mean (95% CIs)	4.72 (4.24; 5.21)	5.81 (5.32; 6.29)	0.004

FRI: functional respiratory imaging, BV5: pulmonary blood volume in vessels with a cross-sectional area of 1.25–<5 mm^2^ (small–medium-sized pulmonary muscular arteries and medium-sized pulmonary veins), BV5–10: pulmonary blood volume in vessels with a cross-sectional area of ≥5–<10 mm^2^ (large elastic segmental pulmonary arteries and medium-sized pulmonary veins), BV10–140: pulmonary blood volume in vessels between ≥10 and 140 mm^2^ (large elastic segmental pulmonary arteries and large pulmonary veins), IVAW: intrapulmonary volume of airway, IVLOBE: intrapulmonary lobar volume. Parameters are reported as age-adjusted means and 95% confidence intervals (CIs).

**Table 4:** Demographic, lung functional test, and quality of life questionnaire parameters of the fully, partially, and non-vaccinated long COVID patients.

	All Long COVID Patients	Fully Vaccinated Long COVID Patients (n = 9)	Partially Vaccinated Long COVID Patients (n = 10)	Non-Vaccinated Long COVID Patients (n = 11)	*p* Value
Age (years)	44.9 ± 12.6	41.2 ± 11.6	47.5 ± 9.7	39.0 ± 13.3	0.567
Male, n (%)	11 (36.7%)	2 (22.2%)	5 (50.0%)	4 (36.4%)	0.233
BMI (kg/m^2^)	25.9 ± 4.3	26.0 ± 3.2	25.7 ± 4.0	26.3 ± 4.3	0.971
Arterial hypertension, n (%)	6 (20.0%)	2 (22.2%)	1 (10.0%)	3 (27.3%)	0.602
Hypercholesterolemia, n (%)	8 (26.7%)	1 (11.1%)	3 (30.0%)	4 (36.4%)	0.428
Diabetes mellitus, n (%)	1 (3.3%)	1 (11.1%)	0 (0%)	0 (0%)	0.299
Smoking, n (%)	3 (10.0%)	1 (11.1%)	1 (10.0%)	1 (9.1%)	0.989
FVC (L)	3.72 ± 0.90	3.48 ± 0.47	4.19 ± 0.68	3.46 ± 0.64	0.170
FEV1 (L)	2.89 ± 0.74	2.87 ± 0.41	3.05 ± 0.49	2.79 ± 0.56	0.473
PEF (L/s)	7.69 ± 1.39	7.72 ± 1.10	8.50 ± 0.95	7.19 ± 0.88	0.171
Number of COVID-19 infections, n (%)	1.03 ± 0.18	1.11 ± 0.33	1 ± 0	1 ± 0	0.322
Number of outpatient visits	3.03 ± 1.52	3.44 ± 1.24	3.30 ± 1.77	2.45 ± 1.44	0.287
Number of organs assigned to the symptoms	4.37 ± 2.24	3.56 ± 2.19	4.90 ± 2.60	4.55 ± 1.92	0.416
Number of symptoms	5.53 ± 3.13	5.22 ± 3.96	5.90 ± 3.35	5.45 ± 2.34	0.897
Time between COVID-19 infection and lung CT (days)	283 ± 158	375 ± 156	256 ± 130	233. ± 162	0.104
Waiting time for first outpatient visit (days)	221 ± 128	255 ± 147	198 ± 125	188 ± 113	0.470
Higher education, n (%)	18 (60.0%)	4 (44.4%)	7 (70.0%)	7 (63.6%)	0.643
Employment status, n (%)	25 (83.3%)	7 (77.8%)	9 (90.0%)	9 (81.8%)	0.764
Anti-SARS-CoV2 spike titer (BAU/mL)	1492 ± 861	2174 ± 861 *	1684 ± 1101	824 ± 1158 *	0.045
VAS scale (range 0/worst to 100/best)	58.1 ± 18.3	71.4 ± 11.1 *†	54.4 ± 19.2 †	50.5 ± 17.5 *	0.023
Resting dyspnea (score 1/none to 10/worst)	1.93 ± 1.86	1.11 ± 1.69	1.60 ± 1.58	2.91 ± 1.92	0.072
Exercise-induced dyspnea (score 1/none to 10/worst)	4.59 ± 3.3	2.44 ± 2.07 *	4.60 ± 2.50	6.50 ± 3.81 *	0.02
Cognitive dysfunction, n (%)	23 (76.7%)	6 (66.7%)	8 (80.0%)	9 (81.8%)	0.612
Cough, n (%)	12 (40.0%)	3 (33.3%)	5 (50%)	4 (36.4%)	0.725

BMI: body mass index, FVC: forced vital capacity, FEV1: forced expiratory volume in 1 s, PEF: peak expiratory flow, VAS: visual analog scale for self-reported health status. Continuous variables are presented as mean ± standard deviation (SD), and categorical variables are presented as numbers (percentages). Comparisons among the three vaccination subgroups were performed using one-way analysis of variance (ANOVA) with Tukey’s post hoc test for normally distributed variables or the Kruskal–Wallis test for non-normally distributed variables. Categorical variables were compared using Pearson’s χ^2^ test. * indicates a significant difference (*p* < 0.05) between the fully vaccinated and non-vaccinated subgroups; † indicates a significant difference (*p* < 0.05) between the fully vaccinated and partially vaccinated subgroups.

**Table 5:** Laboratory parameters of the fully, partially, and non-vaccinated long COVID patients.

Routine Clinical Lab Data	All Long COVID Patients n = 30	Fully Vaccinated Long COVID Patients (n = 9)	Partially Vaccinated Long COVID Patients (n = 10)	Non-Vaccinated Long COVID Patients (n = 11)
Hematology parameter				
Hgb (g/dL)	13.2 ± 2	13.2 ± 0.8	15.2 ± 1.3	13.5 ± 1.3
Platelet (G/L)	254.1 ± 54.4	310.7 ± 96.8	238.2 ± 46.0	255.8 ± 46.6
Leukocyte (G/L)	6.1 ± 1.4	7.5 ± 2.1	6.5 ± 2.1	6.1 ± 1.1
Creatinine (mg/dL)	0.8 ± 0.1	0.80 ± 0.14	0.89 ± 0.18	0.77 ± 0.1
Albumin (g/L)	47.6 ± 2.1	48.3 ± 2.2	48.3 ± 3.3	45.3 ± 2.1
SGOT (U/L)	24.5 ± 8.1	19.0 ± 3.1	28.4 ± 6.9	24.8 ± 10.8
SGPT (U/L)	26.3 ± 18.6	24.6 ± 10.7	38.9 ± 27.6	26.9 ± 14.1
Eisen. (µg/dL)	79.2 ± 28.3	80.0 ± 42.5	80.0 ± 31.9	85.6 ± 30.3
TSH (µIU/mL)	1.2 ± 0.43	1.14 ± 0.25	1.39 ± 0.21	1.33 ± 0.65
Coagulation parameter				
Prothrombin time (%)	96.7 ± 12	96.3 ± 14.6	100.6 ± 10.4	93.3 ± 15.2
INR	1 ± 0.1	1.03 ± 0.06	1.02 ± 0.04	1.03 ± 0.08
aPTT (s)	34.2 ± 4	32.8 ± 2.5	36.3 ± 6.5	35.0 ± 3.3
Fibrinogen (mg/dL)	322.2 ± 47.7	341.8 ± 63.3	330.0 ± 40.8	312.2 ± 57.9
D-dimer (µg/mL)	0.36 ± 0.1	0.40 ± 0.13	0.61 ± 0.44	0.42 ± 0.11
vWF antigen (%)	115.2 ± 39.5	131 ± 53.7	110.0 ± 40.2	119.2 ± 34.3
Cardiological parameter				
Troponin T (ng/L)	7.8 ± 1.9	0 ± 0	9.0 ± 0	7.5 ± 2.4
Creatine kinase (U/L)	104.6 ± 35.3	87.2 ± 27.4	117.6 ± 42.6	116.7 ± 76.9
NT-proBNP (pg/mL)	45.5 (15.6; 71.4)	50.0 (12.7; 72.6)	33.3 (15.2; 71.1)	55.3 (22.0; 75.4)
Inflammatory parameter				
C-reactive protein (mg/dL)	0.24 ± 0.19	0.38 ± 0.55	0.35 ± 0.47	0.26 ± 0.22
LDH (U/L)	170.7 ± 42.9	170.8 ± 37.1	174.7 ± 59.1	177.5 ± 45.0
Ferritin (µg/L)	129.5 ± 136.4	76.2 ± 77.6	125.0 ± 61.4	90.8 ± 56.3
Transferrin (mg/dL)	277.7 ± 41.3	339.3 ± 72.1	273.3 ± 33.7	264.3 ± 48.7
Transferrin saturation (%)	20.3 ± 8.1	18.0 ± 11.5	22.74 ± 7.8	22.6 ± 11.2
Interleukin-6 (pg/mL)	2.00 (0; 3.26)	1.70 (0; 2.19)	2.63 (1.19; 3.68)	2.09 (0; 3.00)
Procalcitonin (ng/mL)	0.04 ± 0.02	0.01 ± 0.02	0.05 ± 0.03	0.03 ± 0.01

Hgb: hemoglobin, SGOT: serum glutamic–oxaloacetic transaminase, SGPT: serum glutamic pyruvic transaminase, TSH: thyroid-stimulating hormone, INR: international normalized ratio, aPTT: activated partial thromboplastin time, vWF: von Willebrand factor, NT-proBNP: N-terminal pro-brain natriuretic peptide, LDH: lactate dehydrogenase. Data are presented as mean ± standard deviation (normal distribution) or median with interquartile ranges (skewed distribution).

## Data Availability

The original contributions presented in this study are included in the article/Supplementary Materials. Further inquiries can be directed to the corresponding author.

## Supplementary Materials

The following supporting information can be downloaded at https://www.mdpi.com/article/10.3390/medsci14030413/s1, Supplementary Methods: Chest CT imaging; Table S1: Additional statistical results of the age-adjusted FRI parameter; Table S2: Functional respiratory imaging (FRI) parameters of the fully, partially and non-vaccinated long COVID patients.

## Abbreviations

The following abbreviations are used in this manuscript:

BMI	Body mass index
BV10	Pulmonary vascular volume in vessels between ≥10 and 140 mm^2^ in cross-sectional area
BV5–10	Pulmonary vascular volume in vessels between ≥5 and <10 mm^2^ in cross-sectional area
BV5	Pulmonary vascular volume in vessels between 1.25 and <5 mm^2^ in cross-sectional area
COPD	Chronic obstructive pulmonary disease
CT	Computed tomography
FRI	Functional respiratory imaging
IVAW	Intrathoracic airway volume
IVLOBE	Intrapulmonary lobar volume of air
LL	Left lobes
LLL	Left lower lobe
LUL	Left upper lobe
PASC	Post-acute sequelae of SARS-CoV-2 infection
POSTCOV	Post-COVID study
RL	Right lobes
RLL	Right lower lobe
RML	Right middle lobe
RUL	Right upper lobe
