# Supplementary material for: Beneficial Effect of COVID-19 Vaccination on Decreased Pulmonary Vascular and Airway Volumes of Patients with Long-COVID Syndrome
Source: Med Sci (Basel). 2026 Jul 22;14(3):413. doi: 10.3390/medsci14030413 (PMC13413875; doi:10.3390/medsci14030413)
Supplement: Supplementary file 1 [file medsci-14-00413-s001.zip › medsci-4326891-supplementary.pdf]

Beneficial Effect of COVID-19 Vaccination on Decreased Pulmonary Vascular and Airway Volumes of Patients with Long-COVID Syndrome

Supplementary File

Supplementary Methods

Chest CT imaging

Briefly, patients were examined in the supine position with arms elevated above the head, and images were obtained in the craniocaudal direction during a single breath-hold at full inspiration. Scans were performed using a dual-source CT system (Siemens SOMATOM Drive).

Detailed imaging acquisition parameters—including collimation, focal spot size, tube voltage, reference tube current, CARE keV IQ level, rotation time, pitch factor, and syngo CT VB20B software—as well as reconstruction parameters (slice thickness, increment, reconstruction plane, kernel, matrix size, iterative reconstruction technique, and field of view) have been reported previously by our radiology group (12). Non-contrast-enhanced CT was used for structural lung assessment. To exclude chronic pulmonary embolism, intravenous iodinated contrast material (60–100 mL) was administered at an injection rate of 3.5 mL/s, followed by a saline flush, with image acquisition and reconstruction performed according to international guideline recommendations (19).

Supplementary Results

Supplementary Table S1. Additional statistical results of the age-adjusted FRI parameter

Age-adjusted FRI parameter statistics	Adjusted mean difference	Beta regression coeffficient	Levene Test
BV5 mL	15.6 (p=0.015)	-0.122 mL/year (-0.67; 0.427; p=0.659)	p=0.308
BV5-10 mL	0.483 (p=0.799)	-0.30 mL/year (-0.196; 0.136; P=0.717)	p=0.189
BV10-140 mL	27.29 (p<0.001)	-0.075 mL/year (-0.403; 0.254; p=0.650)	p=0.380
IVAW mL	15.03 (p=0.004)	-0.179 mL/year (-0.619; 0.261; p=0.419)	p=0.039
IVLOBE L	1.083 (p=0.004)	-0.004 L/year (-0.036; 0.028; p=0.812)	p=0.259

BV5: pulmonary blood volume in vessels with a cross-sectional area of 1.25 - <5mm^2, BV5-10: pulmonary blood volume in vessels with a cross-sectional area of ≥5 - <10mm^2, BV10-140: pulmonary blood volume in vessels between ≥10 and 140 mm^2, IVAW: intrapulmonary volume of airways, IVLOBE: intrapulmonary lobar volume.

Supplementary Table S2. Functional respiratory imaging (FRI) parameters of the fully, partially and non-vaccinated long COVID patients.

FRI Parameter	Fully vaccinated (n=9)	Partially vaccinated (n=10)	Non-vaccinated (n=11)	P value
BV5 LLL (mL)	32.2±8.0	28.6±4.8	27.7±4.7	0.219
BV5 LUL (mL)	27.2±4.3	23.6±3.8	23.5±2.7	0.056
BV5 RLL (mL)	**37.8±7***	32.0±6.3	**30.8±5.3***	**0.046**
BV5 RML (mL)	9.0±2.2	9.4±2.9	8.3±3.0	0.683
BV5 RUL (mL)	**25.2±5.8*+**	**20.0±4.1+**	**20.4±2.9***	**0.026**
BV5 UL (mL)	61.4±11.8	53.0±8.9	52.2±5.7	0.059
BV5 LL (mL)	70.0±14.9	60.6±10.7	58.5±9.7	**0.093**
BV5 TOTAL (mL)	**131.4±21.9***	113.6±15.8	**110.7±13.9***	**0.029**
BV5 10 LLL (mL)	10.8±2.2	8.9±1.3	9.5±2.0	0.087
BV5 10 LUL (mL)	8.4±2.2	6.9±1.3	7.6±1.8	0.211
BV5 10 RLL (mL)	11.1±2.3	9.3±1.7	10.0±2.2	0.187
BV5 10 RML (mL)	2.8±0.4	2.9±0.8	2.8±0.6	0.928
BV5 10 RUL (mL)	6.7±1.8	5.6±1.7	6.2±1.8	0.442
BV5 10 UL (mL)	17.8±4.0	15.4±3.4	16.5±3.7	0.364
BV5 10 LL (mL)	22.0±4.5	18.2±2.8	19.5±4.1	0.120
BV5 10 TOTAL (mL)	39.8±7.7	33.6±5.3	36.1±7.3	0.158
BV10 LLL (mL)	11.2±3.5	9.0±2.3	10.5±4.8	0.442
BV10 LUL (mL)	8.5±3.5	6.4±2.4	8.0±3.9	0.361
BV10 RLL (mL)	11.4±3.3	8.9±2.6	9.9±5.1	0.393
BV10 RML (mL)	3.1±1.0	2.8±1.2	3.3±1.2	0.581
BV10 RUL (mL)	6.5±2.9	4.9±2.2	5.4±2.4	0.365
BV10 UL (mL)	18.2±7.2	14.1±5.5	16.6±6.9	0.403
BV10 LL (mL)	22.6±6.7	17.9±4.6	20.3±9.7	0.410
BV10 TOTAL (mL)	40.7±13.2	32.0±9.5	37.0±16.5	0.381
IVAW RUL (mL)	2.56±1.61	1.53±0.52	2.02±1.14	0.171
IVAW RML (mL)	1.25±0.93	0.79±0.34	0.91±0.64	0.318
IVAW RLL (mL)	4.03±2.91	2.40±1.27	2.88±2.18	0.270
IVAW LUL (mL)	2.80±1.96	1.71±0.62	2.10±1.20	0.223
IVAW LLL (mL)	3.96±2.57	2.30±0.99	2.56±1.69	0.159
IVAW CENTRAL (mL)	31.26±13.45	25.26±6.23	26.79±12.55	0.492

IVAW UL (mL)	6.61±4.37	4.03±1.39	5.03±2.92	0.204
IVAW LL (mL)	7.89±5.47	4.70±2.20	5.43±3.74	0.206
IVAW DISTAL (mL)	14.51±9.71	8.74±3.51	10.46±6.57	0.198
IVAW TOTAL (mL)	45.77±20.16	34.0±8.91	37.25±17.05	0.273
IVLOBE LLL (L)	1.16±0.4	1.04±0.26	1.10±0.45	0.800
IVLOBE LUL (L)	1.23±0.38	1.00±0.17	1.12±0.41	0.344
IVLOBE RLL (L)	1.33±0.39	1.12±0.31	1.19±0.51	0.549
IVLOBE RML (L)	0.45±0.13	0.46±0.23	0.43±0.17	0.93
IVLOBE RUL (L)	0.99±0.33	0.80±0.15	0.88±0.37	0.392
IVLOBE UL (L)	2.68±0.82	2.21±0.31	2.44±0.92	0.399
IVLOBE LL (L)	2.49±0.77	2.17±0.55	2.29±0.96	0.666
IVLOBE TOTAL (L)	**5.51±1.19***	4.38±0.74	**4.18±1.36***	**0.050**

FRI: functional respiratory imaging, BV5: pulmonary blood volume in vessels with a cross-sectional area of 1.25- <5mm^2 (small-medium sized pulmonary muscular arteries and medium-sized pulmonary veins), BV5-10: pulmonary blood volume in vessels with a cross-sectional area of ≥5 - <10mm^2 (large elastic segmental pulmonary arteries and medium-sized pulmonary veins), BV10-140: pulmonary blood volume in vessels between ≥10 and 140 mm^2 (large elastic segmental pulmonary arteries and large pulmonary veins), IVAW: intrapulmonary volume of airways, IVLOBE: intrapulmonary lobar volume. P values represent ANOVA statistics; Post-hoc comparison with Tukey test between the groups; *p<0.05 between fully vaccinated vs non-vaccinated; +p<0.05 between fully vaccinated vs partially vaccinated; Parameters are reported as mean ± standard deviation.
